# Dual Benefits of Calcium Hydroxyapatite Filler: A Prospective Study on Midface Volume Restoration and Skin Quality Enhancement

**DOI:** 10.1111/jocd.70265

**Published:** 2025-05-29

**Authors:** Ji Yeon Hong, Kui Young Park

**Affiliations:** ^1^ Department of Dermatology Chung‐Ang University College of Medicine Seoul Korea

**Keywords:** calcium hydroxyapatite, dermal filler, facial rejuvenation, skin booster, volumization

## Abstract

**Background:**

Calcium hydroxyapatite (CaHA) fillers are widely used for soft‐tissue augmentation due to their volumizing properties and biostimulatory effects. However, clinical evidence supporting the dual role of CaHA as both a volumizer and a skin booster remains limited.

**Objectives:**

To evaluate the efficacy of CaHA filler (VoLassom) in midface volume restoration and assess its effects on skin barrier function, hydration, and elasticity over 24 weeks.

**Methods:**

Fifteen participants aged 19–70 years with moderate midface volume deficiency (Midface Volume Deficiency Scale [MFVDS] grade ≥ 3) received CaHA filler injections. Clinical assessments included MFVDS scoring, the Global Aesthetic Improvement Scale (GAIS), and patient satisfaction ratings at baseline and at weeks 2, 4, and 24. Instrumental evaluations of transepidermal water loss (TEWL), corneometer‐based hydration, and cutometer‐measured elasticity (R2, R5, and R7) were conducted at all timepoints.

**Results:**

MFVDS scores significantly decreased from baseline to weeks 2, 4, and 24, demonstrating sustained volume restoration. GAIS scores remained high, with all participants rated as significantly improved (5.0) at weeks 2 and 4, though slightly reduced at week 24 (4.53 ± 0.52). Patient satisfaction remained consistently high. TEWL significantly decreased at weeks 4 and 24 (*p* < 0.01), indicating improved skin barrier function. Corneometer measurements showed significant hydration improvements at all timepoints (*p* < 0.05 and *p* < 0.001, respectively). Skin elasticity, measured by R2, R5, and R7, significantly increased at weeks 4 and 24 (*p* < 0.01), suggesting enhanced skin firmness.

**Conclusions:**

CaHA filler demonstrated effective and sustained volume restoration while significantly improving skin hydration, barrier function, and elasticity. These findings suggest that CaHA functions not only as a volumizer but also as a skin booster, offering a comprehensive approach to facial rejuvenation. Nevertheless, further studies with larger cohorts are warranted to validate these results.

## 1 Introduction

Calcium hydroxyapatite (CaHA) fillers are widely recognized for their volumizing properties in soft tissue augmentation. Unlike hyaluronic acid fillers, which primarily provide volume through hydrophilic expansion, CaHA offers a dual benefit. They provide immediate structural support and promote long‐term collagen production. CaHA microspheres act as a scaffold, stimulating fibroblast activity and promoting neocollagenesis, thereby contributing to sustained volume enhancement and improved skin quality over time [1].

Beyond volumization, CaHA fillers have recently garnered attention for their ability to improve overall skin quality. These effects are attributed to their capacity to stimulate collagen and elastin synthesis and support extracellular matrix remodeling, which enhances skin hydration, elasticity, and barrier function [2, 3, 4]. Clinical studies have reported improved dermal density and reduced transepidermal water loss (TEWL), further suggesting their potential in mitigating age‐related epidermal dysfunction [5].

Given this gap, this study aimed to evaluate the efficacy of CaHA fillers not only in restoring midface volume but also in improving skin hydration, barrier function, and elasticity. By integrating clinical assessment scales with objective biophysical measurements, this study provides comprehensive insights into the potential of CaHA fillers as skin boosters, offering a more holistic approach to aesthetic and dermatological treatments.

## 2 Materials and Methods

### 2.1 Ethics Statement

The Institutional Review Board (IRB) approved this study (IRB approval number: 2306‐010‐558), ensuring compliance with the Declaration of Helsinki guidelines. Informed consent was obtained from all participants before their enrollment.

To ensure participant confidentiality, all clinical photographs were de‐identified and coded using anonymized subject IDs. These images, along with all other personal data, were stored in a password‐protected and access‐restricted database available only to the study investigators. Participants were explicitly informed about the use of their images for research and publication purposes, and separate written consent was obtained for photography and image use.

### 2.2 Study Design and Participants

This study evaluated the volumizing effects and skin‐boosting benefits of CaHA filler (VoLassom, CGBio Inc., Seoul, Korea) in 15 participants who met the inclusion criteria. A single board‐certified dermatologist administered all filler injections. The study included four visits: baseline (procedure day) and follow‐ups at 2, 4, and 24 weeks. Clinical assessments, participant satisfaction evaluations, and adverse event monitoring were conducted at each visit. All clinical photographs used for GAIS and volumetric evaluations were anonymized prior to review by independent evaluators.

### 2.3 Inclusion and Exclusion Criteria

Participants were healthy adult women aged 19–70 years with volume deficiencies in the zygomaticomalar, anteromedial cheek, or submalar regions. Clinical evaluation confirmed symmetrical midface volume loss graded as moderate (grade 3) or higher on the Midface Volume Deficiency Scale (MFVDS). Only individuals seeking temporary volume restoration who agreed not to undergo other aesthetic procedures—including fillers, botulinum toxin injections, laser treatments, chemical peels, or cosmetic surgery—during the study period were included. Exclusion criteria included the use of anticoagulant or antiplatelet therapy within 10 days before or 3 days after the procedure, a history of facial plastic surgery, tissue grafts, permanent or semi‐permanent fillers, or injections of hyaluronic acid or CaHA fillers within the past 12 months. Participants with active skin infections, untreated wounds, tumors, or a history of radiation therapy in the treatment area were also excluded. Additional exclusion criteria included hypersensitivity to hyaluronic acid, lidocaine, or amide‐type local anesthetics; pregnancy; lactation; participation in another clinical trial within the past 4 weeks; history of anaphylaxis; severe allergies; or systemic diseases that could affect study outcomes.

### 2.4 Injection Procedure

The procedure was performed using a 27G cannula to ensure controlled and precise placement while minimizing trauma. All injections were deliberately administered into the subdermal plane, as our study aimed to assess the effects of undiluted CaHA filler delivered at this depth. The injection sites included the zygomatic region and the anterior‐medial cheek, where up to 0.5 mL of filler was administered per side. Additionally, the subzygomatic area received up to 1.0 mL per side as needed. The selected sites were based on well‐established midface aesthetic units and key volume‐deficient areas commonly treated in clinical practice to restore youthful contour and projection [6]. The total volume per participant did not exceed 4.0 mL for both sides of the face, as determined by the investigator's clinical experience and prior reports demonstrating that moderate volumes can safely achieve optimal outcomes while minimizing the risk of overcorrection or nodularity [1]. Following the injection, gentle massage was applied to the treated areas to ensure even distribution of the filler and achieve a natural contour. All injections were performed by an experienced dermatologist, following aseptic techniques to minimize the risk of complications.

### 2.5 Outcome Measures

The primary endpoint was the effectiveness and durability of volume enhancement, assessed through clinical evaluation and serial photography at baseline, immediately after the procedure, and at 2, 4, and 24 weeks postoperatively. Secondary endpoints included improvements in skin hydration, barrier function, and elasticity, measured by TEWL, corneal hydration, and cutometry‐derived elasticity indices (R2, R5, and R7) at each follow‐up visit. These parameters were selected based on prior validation in dermatologic research as reliable markers of skin firmness (R2), net elasticity (R5), and biological elasticity (R7).

### 2.6 Midface Volume Deficiency Scale (MFVDS) Assessment

MFVDS assessments were conducted by both the investigator and an independent, blinded evaluator at each visit to measure midface volume improvement. Prior to the study, both evaluators were trained to standardize scoring and minimize inter‐rater variability. The MFVDS has been previously validated as a reliable scale for assessing midface volume deficiency in clinical studies. Participants were graded on a five‐point scale: grade 1 (minimal volume loss), grade 2 (mild volume loss), grade 3 (moderate volume loss), grade 4 (severe volume loss), and grade 5 (extreme volume loss). Lower scores indicated mild hollowing, whereas higher scores reflected significant volume depletion. Changes in these scores over time provided insight into the effectiveness of the filler in restoring facial volume.

### 2.7 Global Aesthetic Improvement Scale (GAIS) Evaluation

GAIS assessments were conducted by two independent dermatologists in a blinded manner, who evaluated pre‐ and post‐treatment images. Participants were graded on a five‐point scale: grade 1 (worsening), grade 2 (slight worsening), grade 3 (no change), grade 4 (slight improvement), and grade 5 (significant improvement). Evaluations were conducted separately for the left and right sides of the face, with the overall score calculated as the mean of both sides.

### 2.8 Participant Satisfaction Assessment

Participants self‐reported their overall satisfaction with the treatment outcome on a five‐point scale: grade 1 (dissatisfied), grade 2 (slightly dissatisfied), grade 3 (neutral), grade 4 (slightly satisfied), and grade 5 (highly satisfied).

### 2.9 Adverse Event Monitoring

Adverse events were documented immediately after the procedure and at each follow‐up visit, based on physician evaluation and patient‐reported symptoms. The onset date, resolution date, severity, causality, and necessary interventions were recorded for each case.

### 2.10 Statistical Analysis

Quantitative data were analyzed using paired t‐tests to evaluate changes between baseline and each post‐treatment time point (2, 4, and 24 weeks) for all continuous outcome variables, including transepidermal water loss (TEWL), corneometer hydration, and cutometer elasticity indices (R2, R5, R7). To correct for multiple comparisons across time points, Bonferroni correction was applied. Statistical significance was set at *p* < 0.05. All statistical analyses were two‐tailed and conducted using SPSS Statistics version 26 (IBM Corp., Armonk, NY, USA).

## 3 Results

All 15 participants initially enrolled in the study completed the full 24‐week follow‐up period. There were no protocol deviations or participant withdrawals. Furthermore, no missing data were recorded for any of the clinical assessments or instrumental measurements, allowing for a complete case analysis across all outcome measures.

### 3.1 MFVDS Evaluation

**FIGURE 1:**
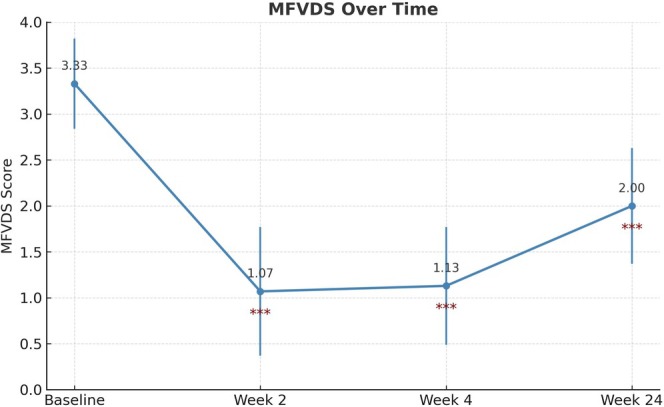
Changes in Midface Volume Deficiency Scale (MFVDS) scores over time. The mean MFVDS score decreased significantly from baseline (3.33 ± 0.49) to week 2 (1.07 ± 0.70, *p* < 0.001) and week 4 (1.13 ± 0.64, *p* < 0.001), demonstrating substantial volume improvement. At week 24, the MFVDS score increased to 2.00 ± 0.63 but remained significantly lower than baseline (*p* < 0.001), indicating partial volume retention. *y*‐axis: MFVDS score (scale 1–5); *x*‐axis: Time point.

MFVDS scores showed a significant reduction from baseline (3.33 ± 0.49) to week 2 (1.07 ± 0.70, *p* < 0.001), week 4 (1.13 ± 0.64, *p* < 0.001), and week 24 (2.00 ± 0.63, *p* < 0.001), demonstrating effective midface volume restoration (paired t‐test with Bonferroni correction; Table 1, Figure 1). Although some inter‐individual variability was observed, no statistical outliers were detected.

**TABLE 1:** Changes in clinical (MFVDS) and instrumental parameters following CaHA filler treatment. Significant improvements were observed in midface volume, skin barrier function (TEWL), hydration, and elasticity parameters (R2, R5, and R7) at both week 4 and week 24 compared to baseline.

Parameter	Baseline (mean ± SD)	Week 4 (mean ± SD)	Week 24 (mean ± SD)	*p*‐value (BL vs. W4)	*p*‐value (BL vs. W24)
MFVDS (score)	3.33 ± 0.49	1.13 ± 0.64	2.00 ± 0.63	< 0.001	< 0.001
TEWL (g/m^2^/h)	12.22 ± 2.21	10.80 ± 2.30	9.91 ± 3.37	0.0031	0.0039
Hydration (AU)	66.53 ± 9.75	75.71 ± 8.52	75.34 ± 7.67	0.0003	0.012
R2 (AU)	58.44 ± 12.00	64.52 ± 11.74	66.87 ± 13.61	0.0002	0.00004
R5 (AU)	41.32 ± 9.66	46.80 ± 9.18	47.41 ± 9.11	0.0073	0.0026
R7 (AU)	35.80 ± 7.92	41.17 ± 7.26	42.08 ± 8.12	0.0024	0.0015

Abbreviations: AU, arbitrary units; BL, baseline.

### 3.2 GAIS Evaluation

**FIGURE 2:**
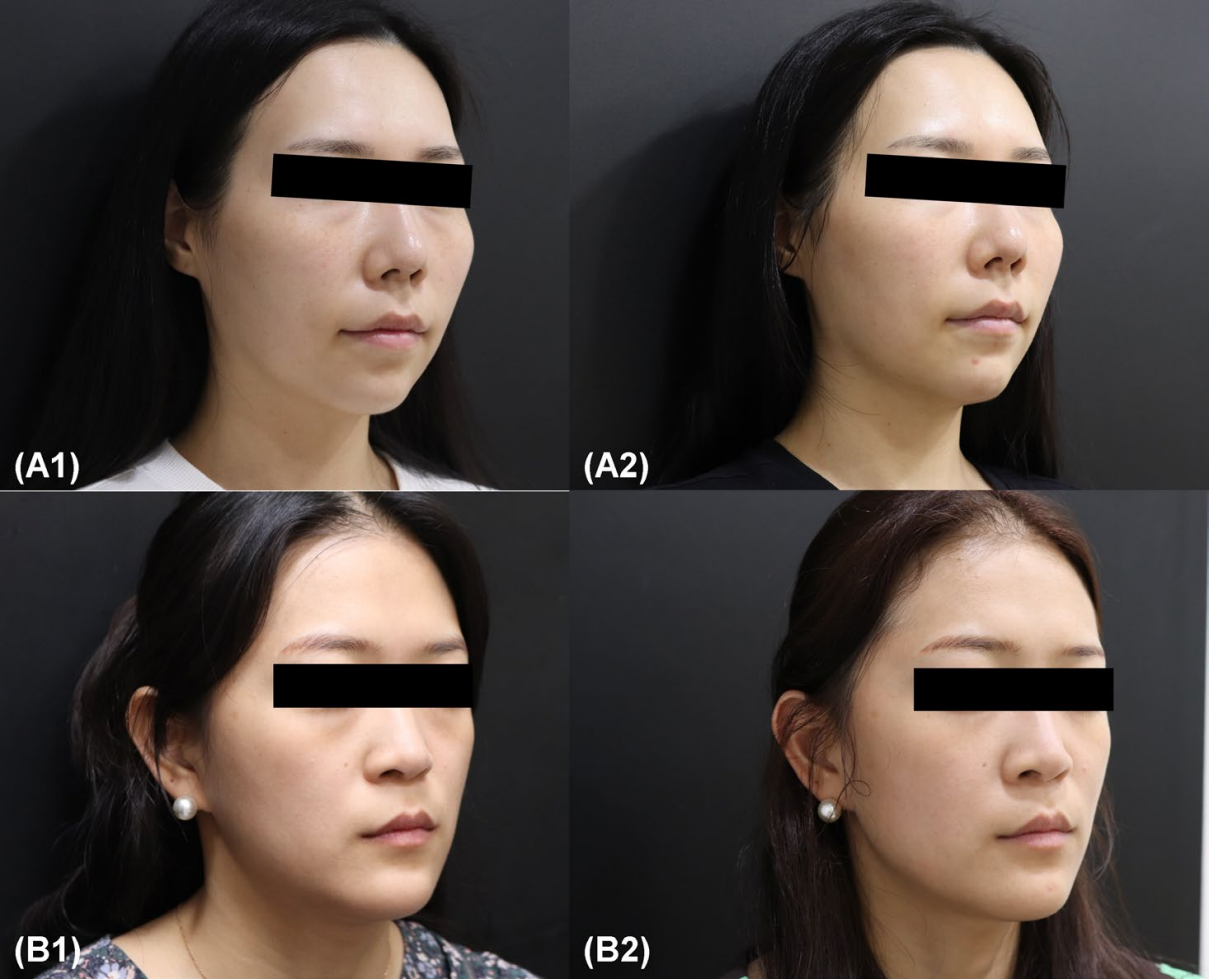
Representative Cases Demonstrating Midface Volume Enhancement After Treatment. Representative cases showing the effects of treatment on midface volume. Case A: (A1) Baseline image before treatment. (A2) Follow‐up image taken 4 weeks post‐treatment, illustrating a noticeable increase in midface volume. Case B: (B1) Baseline image before treatment. (B2) Follow‐up image taken 4 weeks post‐treatment, demonstrating similar volume enhancement in a different patient.

In the GAIS assessment, investigators consistently rated all participants with a score of 5 (significant improvement) at weeks 2 and 4. By week 24, the GAIS score slightly declined to 4.53 ± 0.52, indicating a modest reduction in perceived aesthetic enhancement over time. Representative participant photographs are presented in Figure 2.

### 3.3 Patient Satisfaction Assessment

Patient satisfaction remained high, with mean scores of 5.0 at weeks 2 and 4. A slight decrease to 4.27 ± 0.70 at week 24 reflected a sustained yet mildly diminished perception of treatment efficacy.

### 3.4 Instrumental Measurements

**FIGURE 3:**
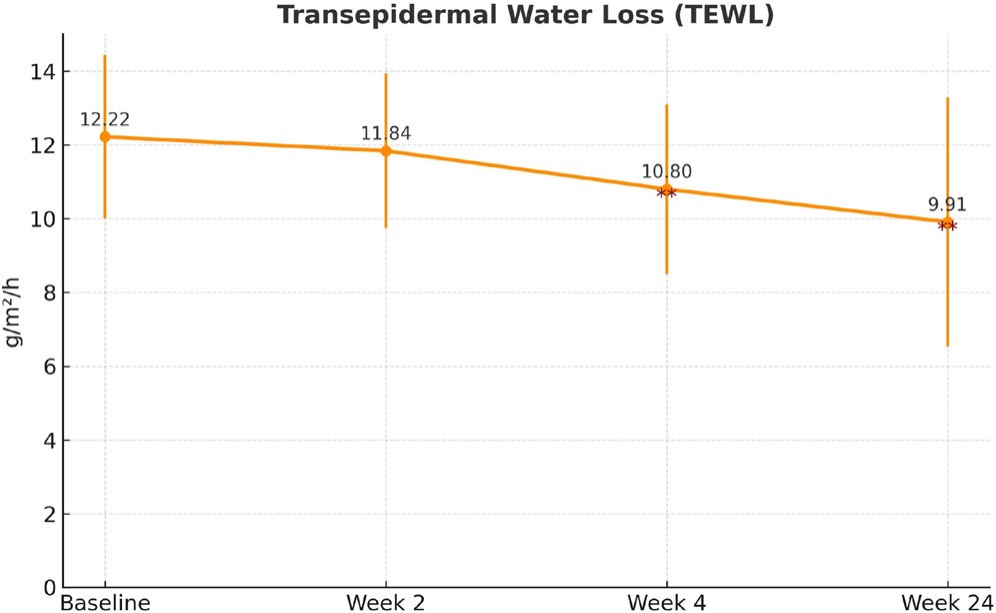
Changes in transepidermal water loss (TEWL) over time. TEWL values showed a decreasing trend from baseline (12.22 ± 2.21 g/h/m^2^) to week 4 (10.80 ± 2.30, *p* < 0.01) and week 24 (9.91 ± 3.37, *p* < 0.01), indicating improved skin barrier function following treatment. *y*‐axis: TEWL (g/m^2^/h); *x*‐axis: Time point.

**FIGURE 4:**
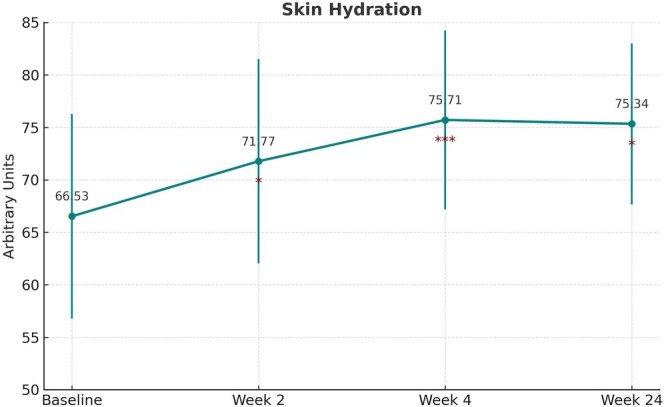
Changes in skin hydration measured by corneometry. Skin hydration improved significantly from baseline (66.53 ± 9.75) to week 2 (71.77 ± 9.74, *p* < 0.05), week 4 (75.71 ± 8.52, *p* < 0.001), and week 24 (75.34 ± 7.67, *p* < 0.05), reflecting increased moisture retention after filler treatment. *y*‐axis: Corneometer units; *x*‐axis: Time point.

**FIGURE 5:**
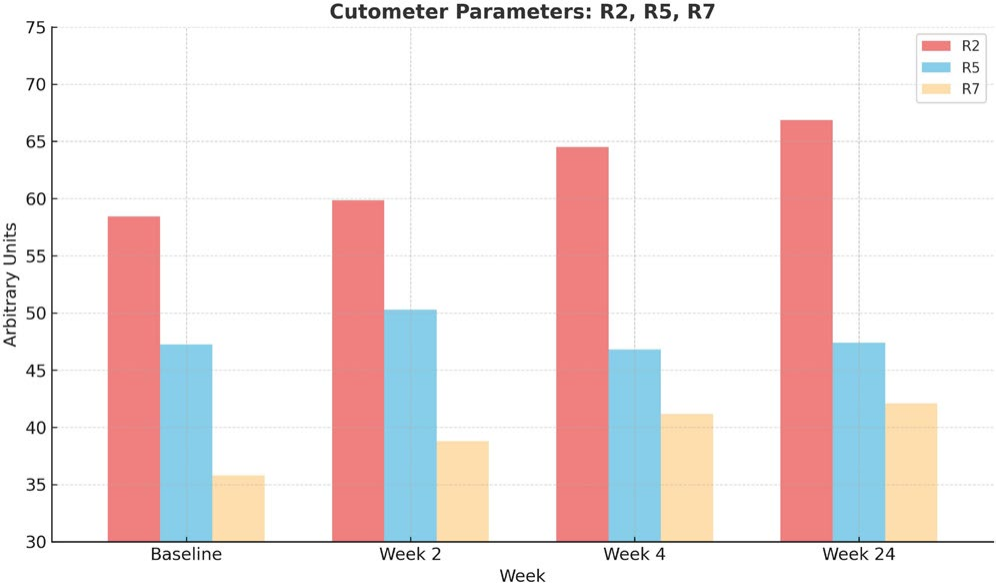
Cutometer measurements of skin elasticity parameters (R2, R5, and R7) at baseline, week 2, week 4, and week 24. Skin elasticity, as represented by R2, R5, and R7, improved over time, with significant increases in R2 at weeks 4 and 24 (*p* < 0.0002 and *p* < 0.00004, respectively), along with significant improvements in R5 and R7, suggesting enhanced skin firmness and resilience following treatment. *y*‐axis: Cutometer units; *x*axis: Time point.

TEWL (g/m^2^/h) decreased significantly from baseline (12.22 ± 2.21) to week 4 (10.80 ± 2.30, *p* = 0.0031) and week 24 (9.91 ± 3.37, *p* = 0.0039; Figure 3). Corneal hydration (arbitrary units, AU) improved significantly at all time points (*p* = 0.0201, *p* = 0.0003, *p* = 0.012, respectively; Figure 4). Cutometer R2 values (AU) increased significantly at week 4 (64.52 ± 11.74, *p* = 0.0002) and week 24 (66.87 ± 13.61, *p* = 0.00004). Additionally, R5 and R7 values improved significantly, further supporting the positive effect of the filler on skin elasticity (*p* < 0.05 at all time points; Figure 5).

### 3.5 Adverse Event Monitoring

No serious adverse events were observed. Mild, transient swelling and redness were reported in some participants, but resolved spontaneously within a few days.

## 4 Discussion

The results of this study provide compelling evidence that CaHA fillers not only offer sustained volume enhancement but also exert notable skin‐boosting effects. The reduction in MFVDS scores over 24 weeks supports previous findings that CaHA fillers provide durable midface volume restoration through a combination of immediate scaffold support and long‐term collagen stimulation [1]. The persistence of volumization at 24 weeks aligns with prior research indicating that CaHA induces neocollagenesis (the formation of new collagen fibers in the skin), leading to long‐lasting structural augmentation [2].

Beyond volumization, our findings also support CaHA's ability to improve hydration, elasticity, and skin barrier function. The significant reduction in TEWL at weeks 4 and 24 suggests enhanced skin barrier integrity, likely mediated by fibroblast stimulation and extracellular matrix remodeling. Previous studies have shown that CaHA increases type I and type III collagen deposition, contributing to improved dermal hydration and resilience [3, 4]. Corneometer findings further supported this, as hydration levels significantly increased following treatment, consistent with reports that CaHA stimulates glycosaminoglycan production, which plays a role in moisture retention [5].

Skin elasticity parameters (R2, R5, and R7) also improved significantly at weeks 4 and 24, indicating enhanced dermal biomechanical function. This aligns with both preclinical and clinical studies reporting that CaHA stimulates elastin synthesis and reinforces dermal architecture [7, 8]. The maintenance of elasticity gains at 24 weeks, despite a mild decline in volumization, suggests a lasting regenerative effect independent of volume alone.

Importantly, while CaHA is commonly diluted and injected superficially for skin rejuvenation, our study demonstrated that undiluted CaHA administered into the subdermal layer led to significant improvements in both volume and skin quality. Unlike most previous studies that focused on either volumization or skin biophysical parameters, we evaluated both domains using objective instrumentation in a single‐session protocol with 24‐week follow‐up. This design offers valuable insight into the intrinsic regenerative potential of CaHA without relying on dilution or superficial injection [6].

Our findings also contribute to the ongoing comparative discussion on biostimulatory fillers. A comparative overview of commonly used fillers—including hyaluronic acid (HA), Poly‐L‐lactic acid (PLLA), polycaprolactone (PCL), and CaHA—is provided in Table 2. HA fillers primarily restore hydration and provide temporary volume with minimal induction of neocollagenesis. While they are commonly injected into the superficial to mid‐dermis for fine lines and contour correction, certain HA fillers are also administered into deep fat compartments or the subdermal plane via cannula, depending on their rheologic properties and treatment objectives [1]. A major advantage of HA fillers is their reversibility using hyaluronidase, which provides an added margin of safety in managing overcorrection or vascular complications [9]. PLLA and PCL stimulate neocollagenesis more robustly but require multiple sessions and have slower onset of action. PLLA, in particular, must be reconstituted prior to use and demands deep injection with post‐treatment massage to reduce the risk of nodule formation. These agents are also not easily reversible once injected [10, 11]. In contrast, CaHA offers a balanced profile of immediate volumization and long‐term collagen stimulation, often achievable in fewer sessions. In this study, undiluted CaHA was administered into the subdermal layer using a blunt cannula to enable controlled delivery and minimize trauma. A systematic review confirmed that non‐HA fillers, including CaHA, are associated with long‐lasting outcomes, high patient satisfaction, and a favorable safety profile when properly injected [12].

**TABLE 2:** Comparison of Common Dermal Fillers in Aesthetic Practice. Summary of characteristics, indications, mechanisms, and clinical considerations for commonly used filler types.

Filler type	Composition	Mechanism of action	Typical indications	Onset and duration	Injection depth	Number of sessions	Adverse events
Hyaluronic Acid (HA)	Cross‐linked HA	Hydration and space‐filling for volumization	Fine lines, lips, tear troughs, mild contouring	Immediate onset; lasts 6–12 months	Superficial to mid‐dermis	1 (with possible touch‐up)	Mild swelling, bruising, Tyndall effect (rare)
Poly‐L‐lactic Acid (PLLA)	Biodegradable PLLA microspheres	Indirect volume via delayed neocollagenesis (via mild inflammatory response)	Facial lipoatrophy, general facial volume loss	Gradual onset (6–12 weeks); lasts 18–24 months	Deep dermis to subcutaneous tissue	2–3 sessions required	Nodules, palpable lumps, delayed granuloma formation
Polycaprolactone (PCL)	PCL microspheres in gel carrier	Collagen stimulation through slow degradation	Midface contouring, jawline definition, hands	Gradual onset (4–8 weeks); lasts up to 24 months	Deep dermis	1–2 sessions	Mild swelling, occasional nodularity
Calcium Hydroxylapatite (CaHA)	CaHA microspheres in aqueous gel carrier	Immediate volumization plus long‐term collagen stimulation	Midface volume restoration, skin quality improvement, nasolabial folds, hands	Immediate and progressive effect; lasts 12–18 months	Deep dermis to subdermis	1 session (may repeat annually)	Edema, erythema, rare nodules or induration

Recent histologic studies indicate that CaHA promotes type I and III collagen production within 3–6 months [13, 14], while PLLA and PCL require longer periods (up to 24 months) for similar effects. Moreover, unlike PLLA, which elicits collagen synthesis via inflammatory pathways, CaHA achieves neocollagenesis with minimal inflammatory response, supporting its superior tolerability and suitability for skin quality improvement [15]. Direct fibroblast–microsphere interaction appears central to this process, with studies reporting type III collagen increase within 24 h and type I collagen increase within 72 h post‐CaHA exposure [16].

Long‐term benefits have also been supported by clinical observations. In a 36‐month prospective study, Wollina and Goldman demonstrated sustained facial rejuvenation following repeated CaHA treatments in elderly women, with some effects lasting up to 5 years [17]. Similarly, a review by Emer and Sundaram compiled evidence from controlled trials and meta‐analyses showing volumization effects extending beyond 30 months, reinforcing CaHA's utility as both a volumizer and scaffold for tissue remodeling [18].

The improvements in hydration, elasticity, and barrier function observed in our study further support CaHA's comprehensive skin‐enhancing properties. These are likely mediated by the combined effects of neocollagenesis, neovascularization, and matrix remodeling [19]. Patient‐reported outcomes—including GAIS and satisfaction scores—remained consistently high, reflecting both objective and subjective benefits. Although a slight decline in GAIS scores was noted at 24 weeks, this coincided with maintenance of biophysical skin improvements, suggesting a dissociation between volume loss and dermal rejuvenation.

Nevertheless, the findings should be interpreted with caution. First, the relatively small sample size (*n* = 16) limits generalizability. Second, the lack of a control group precludes direct comparison with untreated or alternatively treated areas. Third, outcome assessments included subjective scales (GAIS, satisfaction), which may be prone to bias. In addition, the wide age range (19–70 years) may have introduced variability in treatment response, given age‐related differences in collagen dynamics. Future studies should include age‐stratified analyses, blinded evaluators, and control arms to strengthen internal validity. Longer follow‐up and larger cohorts are also warranted to assess long‐term effects and compare CaHA with other biostimulatory fillers.

## 5 Conclusion

This study highlights the dual functionality of CaHA as both a volumizing agent and a biostimulatory skin enhancer, demonstrating clinically and instrumentally measurable improvements in hydration, barrier function, and elasticity. To our knowledge, this is one of the first prospective studies to comprehensively assess the integrated effects of undiluted, subdermally injected CaHA on both midface volume and objective skin quality parameters over a 24‐week period. This adds new insight into CaHA's long‐term regenerative capacity. Despite these promising findings, further randomized controlled trials with larger populations and longer follow‐up durations are needed. Comparative studies with other biostimulatory agents will also be essential to determine the optimal indications, mechanisms, and clinical applications of CaHA in facial rejuvenation.

## Author Contributions

J.Y.H. participated in data collection, analysis, and manuscript drafting. K.Y.P. conceptualized and designed the study, supervised the research process, contributed to data interpretation, and critically revised the manuscript. Both authors have read and approved the final manuscript.

## Conflicts of Interest

K.Y.P. is a consultant for CGBio Inc.

## Data Availability

The data that support the findings of this study are available from the corresponding author upon reasonable request.
